# The Effectiveness of Extracorporeal Shockwave Therapy for Midportion Achilles Tendinopathy: A Systematic Review

**DOI:** 10.7759/cureus.26960

**Published:** 2022-07-18

**Authors:** Kaylem M Feeney

**Affiliations:** 1 Orthopaedics, Bon Secours Hospital Galway, Galway, IRL

**Keywords:** non insertional, midportion, tendinopathy, chronic, shockwave, eswt, tendon, achilles

## Abstract

Achilles tendinopathy is one of the most common lower limb injuries in both athletes and the general population. Despite the plethora of conservative treatment options available for the management of Achilles tendinopathy, as many as one in four patients will go on to require surgery. Extracorporeal shockwave therapy (ESWT) has emerged as a promising treatment option and has been successful in the management of other common musculoskeletal injuries such as plantar fasciitis. However, the evidence for ESWT in the management of Achilles tendinopathy remains inconclusive. Therefore, the aim of this systematic review was to evaluate the current evidence for the use of ESWT in the management of midportion Achilles tendinopathy.

A comprehensive literature search was conducted using the databases MEDLINE (Pubmed), AMED, EMBASE, CINAHL, and CENTRAL. The databases were searched from their inception to December 2021. This was conducted to identify randomised control trials (RCTs) evaluating the effectiveness of ESWT versus control treatment in the management of midportion Achilles tendinopathy.

Following a comprehensive search of the literature, a total of 283 articles were identified. Following the screening of titles and abstracts, 236 articles were excluded. The main reasons for exclusion were the identification of duplicates, non-randomised studies, and the use of ESWT on other pathology. Following the exclusion of 236 articles, 47 articles were retrieved for full-text review. Of these 47 articles, 40 were excluded leaving a total of 7 RCTs eligible for inclusion in this review. There was consistent evidence from 4 RCTs that ESWT is effective in the management of midportion Achilles tendinopathy.

This review suggests that ESWT is a safe and effective modality for treating midportion Achilles tendinopathy as it reduces pain and improves function. The best available evidence suggests that a combination of ESWT with eccentric exercises and stretching may be even more effective than ESWT alone. Further research is required to confirm this and to determine the optimum ESWT treatment protocol.

## Introduction and background

The Achilles tendon is the largest and strongest tendon in the human body [1,2]. Through the force generated by the gastrocnemius and soleus muscles, the primary function of the Achilles tendon is to plantarflex the ankle joint [1]. The Achilles tendon, despite its strength, is one of the most frequently injured tendons in the human body and accounts for approximately 9% of general sports injuries and up to 18% of running injuries [3,4]. Achilles tendon injury, however, is not restricted to those involved in sports and as many as 33% of Achilles tendon injuries occur in sedentary individuals [5].

Achilles tendinopathy encompasses the conditions of Achilles paratenonitis, which describes inflammation of the paratenon surrounding the Achilles tendon, and Achilles tendinosis, which describes tendon fibre degeneration without intratendinous inflammation, which can occur at the insertion (insertional) or in the body of the tendon (midportion or non-insertional) [6-8]. These disorders can only truly be diagnosed through imaging and/or histopathological examination and in clinical practice, therefore, the most appropriate term to use clinically is Achilles tendinopathy [6,8].

The management of Achilles tendinopathy is largely conservative, despite approximately 25%-29% of patients reportedly going on to require surgery for the condition [9,10]. Conservative options described in the literature for the management of midportion Achilles tendinopathy are plentiful and include eccentric loading exercises, heavy slow resistance training, activity modification, non-steroidal anti-inflammatory medications, friction massage, therapeutic ultrasound, orthoses, injection therapy, the use of a night splint, calf stretching, taping, heel lifts, and extracorporeal shockwave therapy (ESWT) [4,7,10-21]. Despite the plethora of options available, there is no general consensus as to the most effective conservative modality for midportion Achilles tendinopathy, though the highest level of evidence in systematic reviews supports an eccentric loading program [4,10,15,16]. Despite a large number of conservative options available, a significant proportion of patients go on to require surgery for their condition [10]. ESWT is of significant interest to health professionals who manage lower limb pathology as it is becoming increasingly utilised in the management of Achilles tendinopathy and has been the focus of several research studies in recent years, despite inconclusive evidence regarding its effectiveness [19-24].

The most recent topical review evaluating the effectiveness of various treatments for midportion Achilles tendinopathy was published in 2020 by Jarin et al. [25]. They concluded that the use of ESWT was well supported in the literature as a second-line treatment for midportion Achilles tendinopathy. Unfortunately, however, their study identified just four [26-29] of the six [24,26-30] randomised controlled trials (RCTs) evaluating the effectiveness of ESWT in the management of midportion Achilles tendinopathy that were available in the literature at the time of their literature search. They also included one case-control study and one prospective cohort study in their review [31,32]. The failure to include the other two RCTs available in the literature at the time of their literature search means that not all of the available evidence was included, potentially affecting the results [24,30]. Unfortunately, they did not assess study quality or perform risk of bias (ROB) assessment of included studies. In addition, since the review by Jarin et al. [25] was published another RCT on this topic has been published [33].

Another recent systematic review and meta-analysis of nonsurgical therapies for the treatment of midportion Achilles tendinopathy was carried out by Rhim et al. in 2020 [34]. They concluded that ESWT could be used alongside eccentric exercises to improve clinical outcomes. However, they only included two of the six available RCTs in the literature at the time of their review [28,29]. Failing to include the other four RCTs evaluating ESWT’s effectiveness for Achilles tendinopathy means that much of the current evidence has not been taken into account in their recommendations.

In contrast to the abovementioned systematic reviews, Punnoose et al. [35] carried out a systematic review and meta-analysis and concluded that ESWT resulted in no significant improvement in pain or function when compared with control, though they only included articles up to their search in 2013 and therefore these recommendations are not current. Therefore, the primary aim of this review was to evaluate and critically appraise the current evidence base from all RCTs for the effectiveness of ESWT in the management of midportion Achilles tendinopathy in adults.

## Review

Methods

A comprehensive literature search was conducted using the databases MEDLINE (Pubmed), AMED, EMBASE, CINAHL, and CENTRAL. The databases were searched from their inception to December 2021. This was conducted to identify RCTs evaluating the effectiveness of ESWT versus control treatment in the management of midportion Achilles tendinopathy. The PICO process was used to identify keywords for the literature search and included search terms such as Achilles, Tendinopathy, ESWT, Shockwave, and Treatment [36]. In addition, The World Health Organisation International Clinical Trials Registry Platform (https://trialsearch.who.int/) and Clinical Trials Registry (https://clinicaltrials.gov/) were searched for ongoing or unpublished RCTs. Finally, reference lists of identified systematic reviews and RCTs were screened for relevant RCTs. 

Study selection

The titles and abstracts of articles identified in the literature search of each database were screened by the first author. Broad review criteria were utilised in the initial screening of abstracts and titles, including keywords such as Achilles, ESWT, and tendinopathy. Following initial screening, the full texts of relevant articles were retrieved and screened by the first author using the narrow search criteria based on the population, intervention, comparator, and outcome measure (PICO) criteria. All RCTs identified in the literature search, from the inception of each database up to the point of the literature search in December 2021, were eligible to be included. Any study that was not a RCT was excluded. RCTs published in a language other than English were excluded. 

Data extraction

The Cochrane Data Extraction and Assessment Template was used by the reviewer to extract all relevant data from each RCT. The first author used the GRADE approach [37] to categorize the level of quality of each included RCT. The GRADE system categorizes the level of quality of a research study as “high,” “moderate,” “low,” or “very low” quality based on several different factors [37].

Methodological quality

The methodological quality of included studies was evaluated independently by the first author using the Cochrane ROB assessment tool [38]. Only RCTs were included in this review in order to ensure the highest quality studies were analysed. RCTs were included regardless of their ROB. Uncontrolled studies, non-randomised studies, systematic reviews, and studies in a language other than English were excluded.

Results

**Figure 1:**
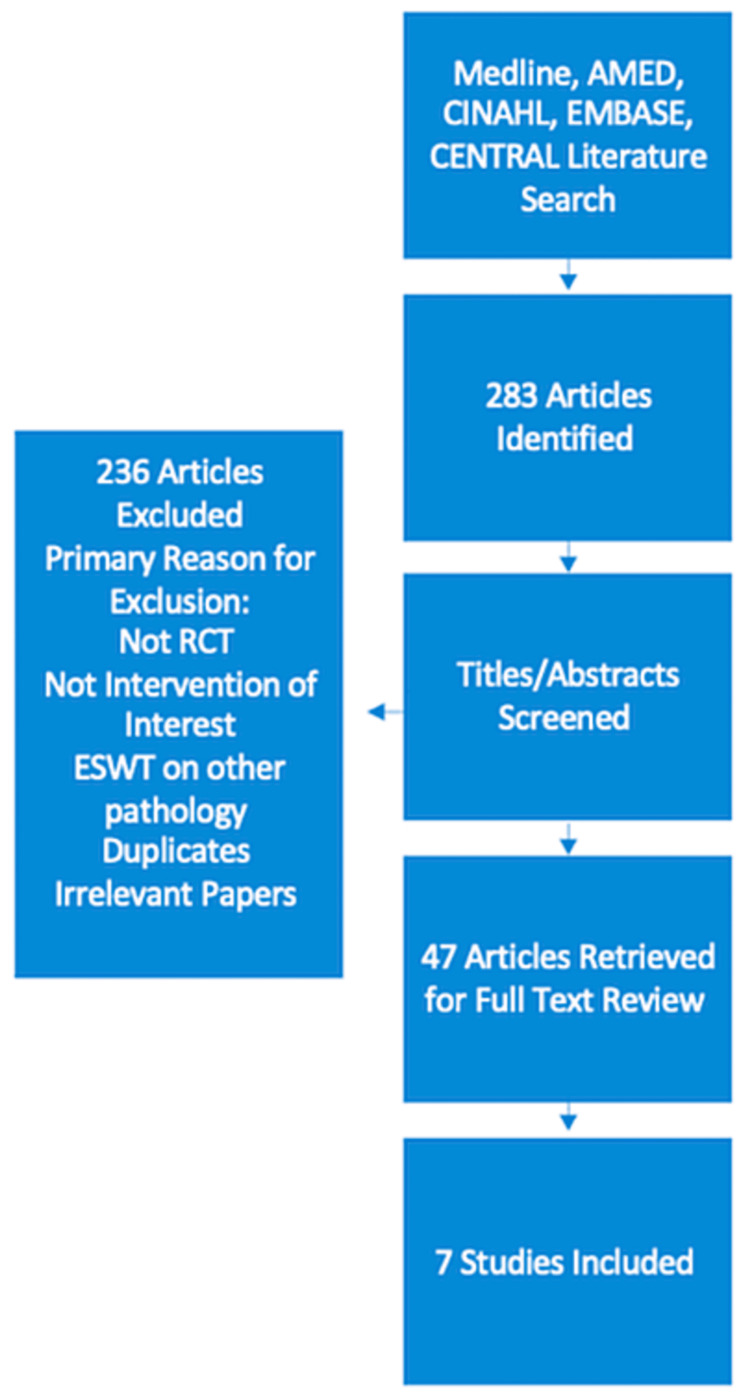
PRISMA flow diagram

Following a comprehensive search of the literature, a total of 283 articles were identified. Following the screening of titles and abstracts, 236 articles were excluded. The main reasons for exclusion were the identification of duplicates, non-randomised studies, and the use of ESWT on other pathology. Following the exclusion of 236 articles, 47 articles were retrieved for full-text review. Of these 47 articles, 40 were excluded leaving a total of 7 RCTs eligible for inclusion in this review. A summary of the PRISMA flow diagram can be found in Figure 1. A summary of included studies can be found in Table 1.

**Table 1:** Summary of included studies ESWT, extracorporeal shockwave therapy; VAS, Visual Analog Scale; FIL, functional index of lower limb activity; EQoL, EuroQoL generalised health status questionnaire; VISA-A, Victorian Institute of Sports Assessment-Achilles questionnaire; NRS, numeric rating scale; AOFAS, American Orthopaedic Foot and Ankle Society score

Study	Groups	Sessions	Patients	Outcome	Follow up (months)	Conclusion
Costa et al. (2005) [26]	ESWT Control (sham ESWT)	3 over 2 months	49 (2 groups)	VAS FIL EQoL-5D and health score Clinical assessment	3 and 12	No support for use of ESWT in the management of chronic tendinopathy.
Rompe et al. (2007) [29]	Eccentric loading ESWT Wait-and-see	3 over 3 weeks	75 (3 groups)	VISA-A Likert scale Load-Induced Pain (NRS)	4	ESWT and eccentric exercises comparable, both superior to wait-and-see group, which was ineffective.
Rasmussen et al. (2008) [30]	Stretching, eccentric exercises and ESWT Stretching, eccentric exercises and sham ESWT	4 over 4 weeks	48 (2 groups)	VAS (walking, on stairs, working, running) AOFAS score	1, 2 and 3	ESWT resulted in a statistically significant improvement in AOFAS but not VAS score. ESWT may supplement standard treatment.
Rompe et al. (2009) [28]	Eccentric loading Eccentric loading + ESWT	3 over 3 weeks	68 (2 groups)	VISA-A Likert scale Load-Induced Pain (NRS)	4 and 12	Eccentric exercises combined with ESWT significantly more effective than eccentric exercises alone (VISA-A score).
Vahdatpour et al. (2018) [24]	Conservative care + ESWT Conservative care + sham ESWT	4 over 4 weeks	43 (2 groups)	VAS AOFAS	1 and 4	ESWT group showed a statistically significant improvement in both VAS and AOFAS score at 4 months but not 1 month.
Abdelkader et al. (2021) [33]	Stretching, eccentric exercises and ESWT Stretching, eccentric exercises and sham ESWT	4 over 4 weeks	50 (2 groups)	VAS VISA-A	1 and 16	Both groups improved significantly. Adding ESWT to eccentric exercises and stretching resulted in statistically significant improvement in VAS and VISA compared to control at both time points.
Gatz et al. (2021) [39]	Physiotherapy and point ESWT Physiotherapy and line ESWT Physiotherapy and sham ESWT	4 over 6 weeks	66 (3 groups)	VISA-A AOFAS Likert scale Roles and Maudsley	1.5 and 6	All groups improved significantly. No statistically significant improvement in either ESWT groups compared with placebo.

Study type and sample size

All studies included were RCTs. The mean sample size in included studies was 57, with a range of 43-75 participants in each study. Five of the included studies carried out a power and sample size calculation, all of which achieved their target sample size [28-30,33,38]. The other two studies did not carry out a power or sample size calculation [24,26].

Demographics

With the exception of one study [24], all included studies had comparable gender distribution. In the study by Vahdatpour et al., females were overrepresented, accounting for 82% and 81% of the intervention and control groups, respectively [24]. The mean age of included participants ranged from 28.3 to 58.7 in all included studies. One study had a significant variation in the mean age of 11 years between treatment and control groups [26]. The mean duration of symptoms of participants prior to study entry varied widely in included studies from 4.3 months [24] to 24 months [39], though no significant intrastudy differences between groups were observed. Two studies did not state the duration of symptoms prior to study entry [30,33]. No other significant variations in baseline demographics were observed in any study. All but one study [30] included a statement regarding ethical approval prior to study initiation.

Diagnostic criteria

Five studies diagnosed midportion Achilles tendinopathy based on history and physical examination [24,26,30,33,39], while two studies confirmed the presence of Achilles tendinopathy by combining history and physical examination with ultrasound findings [28,29].

Intervention - ESWT

Three studies utilised radial ESWT only [28-30], one study utilised a combination of radial and focussed ESWT [24], one study compared radial and focussed ESWT [39], two studies utilised focussed ESWT only [26,33] Three studies administered three ESWT sessions to participants [26,28,29], while the remaining studies administered four sessions [24,30,33,39]. The dose of ESWT administered by Vahdatpour et al. was a combination of 1,500 focussed impulses and 3,000 radial impulses, which was a significantly higher dosage of ESWT than the other studies [24]. Five of the included studies administered 2,000 impulses per treatment [28-30,33,39], while one study administered 1,500 impulses per treatment [26].

Variation was also observed across studies with the frequency (Hz) of ESWT. One study did not state the frequency used [26]. The most common frequency used in three studies was an ESWT frequency of 8Hz [28,29,33]. Vahdatpour et al. [24] utilised a frequency of 2.3Hz (focussed) and 2.1Hz (radial), while Gatz et al. [39] used a frequency of 5Hz. The most significant variation in frequency was in the study by Rasmussen et al. [30], where a frequency of 50Hz was used.

Outcome measures

Four of the included studies utilised the VAS scale to measure the outcome of pain [24,30,33,39], while two studies used the Likert scale [29,39]. The VISA-A score, which is specific to the Achilles tendon, was also used in four studies [28,29,33,39]. Three studies utilised the AOFAS score to assess pain and function [24,30,39]. Costa et al. [26] also utilised the FIL, EQoL-5D, and health score in their study, while Gatz et al. [39] were the only researchers to report the Roles and Maudsley (R&M) score.

Follow up

The length of follow-up across all studies ranged from three to 16 months, with Abdelkader et al. [33] reporting the longest follow-up period of 16 months. Three out of the seven studies reported a follow-up of 12 months or more [26,28,33].

Methodological quality of included studies

The results of the ROB using the Cochrane ROB assessment tool are summarised in Table 2. The ROB assessment determined that two of the included studies met the criteria for a “High Quality” study [30,33] based on the GRADE Approach [37]. The other five studies were downgraded from “High Quality” to “Moderate Quality” due to a high ROB in one domain, or an unclear ROB in a number of domains [24,26,28,29,39]. 

**Table 2:** Summary of cochrane ROB assessment ROB - Risk of Bias

	Gatz et al. (2021) [39]	Abdelkader et al. (2021) [33]	Vahdatpour et al. (2018) [24]	Rompe et al. (2009) [28]	Rasmussen et al. (2008) [30]	Rompe et al. (2007) [29]	Costa et al. (2005)[26]
Random Sequence Generation	?	+	?	+	+	+	+
Allocation Concealment	+	+	?	+	+	+	+
Blinding of Participants and Personnel	+	+	+	–	+	–	–
Blinding of Outcome Assessment	?	+	+	+	+	+	+
Incomplete Data Outcome	+	+	+	+	+	+	+
Selective Reporting	?	+	?	?	+	?	+
Other Bias	?	+	?	+	?	+	+

Summary of effectiveness of ESWT v control

A visual summary of the effectiveness of ESWT versus control is highlighted in Table 3. Of the seven included studies, three studies reported no statistically significant improvement in outcomes with the use of ESWT versus control [24,26,29,39]. Costa et al. [26] found no statistically significant improvement in VAS, FIL, EQoL, and health score with ESWT compared to sham ESWT, while Rompe et al. [29] suggested that both ESWT and eccentric exercises are superior to the wait-and-see group, which was statistically significant. However, the ESWT group was not superior to the eccentric exercise group. Finally, Gatz et al. [39] observed a statistically significant improvement in VISA-A score in all three groups compared to baseline, but their results suggested no significant benefit from point or line ESWT combined with physiotherapy, compared with physiotherapy and sham ESWT.

Four of the included studies observed a statistically significant benefit in the ESWT groups compared with control. Rasmussen et al. [30] observed that stretching combined with eccentric exercises and ESWT resulted in a statistically significant improvement in AOFAS score, but not VAS score, compared to stretching combined with eccentric exercises and sham ESWT. In a subsequent study, Rompe et al. [28] found that a combination of ESWT with eccentric exercises resulted in a statistically significant improvement in VISA-A score, Likert scale, and load-induced pain (NRS) compared with eccentric exercises alone. Finally, Abdelkader et al. [33] determined that both groups (stretching, eccentric exercises, and ESWT) versus stretching, eccentric exercises, and sham ESWT had a statistically significant improvement in VAS and VISA-A scores. Despite the significant improvement in both groups, however, the intervention group had a statistically significant improvement in VAS and VISA-A scores compared with the control group. Finally, Vahdatpour et al. [24] observed a statistically significant improvement in VAS and AOFAS scores in the ESWT group versus the sham ESWT group at the four-month follow-up.

**Table 3:** Summary of effectiveness of ESWT versus control ESWT - Extracorporeal shockwave therapy

Author	Intervention	Control	Statistically Significant Benefit Over Control
Costa et al. (2005) [26]	ESWT	Sham ESWT	No
Rompe et al. (2007) [29]	ESWT OR Eccentric Loading Exercises	Wait-and-see	Yes
Rasmussen et al. (2008) [30]	Stretching, Eccentric Exercises + ESWT	Stretching, Eccentric Exercises + Sham ESWT	Yes (AOFAS) No (VAS)
Rompe et al. (2009) [28]	Eccentric Loading Exercises + ESWT	Eccentric Loading Exercises	Yes
Vahdatpour et al. (2018) [24]	Conservative Care + ESWT	Conservative Care + Sham ESWT	Yes
Abdelkader et al. (2021) [33]	Stretching, Eccentric Exercises + ESWT	Stretching, Eccentric Exercises + Sham ESWT	Yes
Gatz et al. (2021) [39]	Physiotherapy + point OR line ESWT	Physiotherapy + sham ESWT	No

Discussion

The aim of this systematic review was to review the current evidence from all RCTs on the effectiveness of ESWT for the management of midportion Achilles tendinopathy. Following a thorough search of the literature, seven studies met the criteria for inclusion. Overall, four of the seven RCTs included found a statistically significant improvement in outcome measures with the use of ESWT compared to control. Despite the fact that the other three studies observed no statistically significant improvement in outcome measures with the use of ESWT compared to control, each did observe a significant improvement in the ESWT groups from baseline.

While Costa et al. [26] did not observe a statistically significant improvement in outcome measures, their wide confidence intervals suggest that there may still be a clinically significant treatment effect. A larger sample size would have reduced the risk of a type 2 error in this study and may have allowed the researchers to more accurately assess the effectiveness of the intervention.

In an earlier RCT by Rompe et al. [29], they observed a statistically significant improvement in outcome measures in both the ESWT group and eccentric exercises group compared to the wait-and-see group, but no significant benefit in the ESWT group compared to the eccentric exercise group. However, in a follow-up RCT two years later, Rompe et al. [28] found that a combination of ESWT and eccentric exercises resulted in a statistically significant improvement in outcomes measures compared to eccentric exercises alone. These two RCTs suggest that while both ESWT and eccentric exercises are both individually effective in managing midportion Achilles tendinopathy, a combination of both modalities may be even more effective in reducing pain and improving function.

In contrast to the latter study by Rompe et al. [28], Gatz et al. [39] found that physiotherapy plus line or point ESWT resulted in no statistically significant improvement in outcome measures compared to physiotherapy and sham ESWT. Unfortunately, however, the authors did not present the results of two of the four outcome measures (R&M score and Likert scale).

While Vahdatpour et al. [24] did not observe a statistically significant improvement in VAS or AOFAS scores in their one-month follow-up, the ESWT group demonstrated a statistically significant improvement in outcome measures at four-month follow-up, suggesting that ESWT may take a number of weeks before the full effects are observed.

Rasmussen et al. [30] observed a statistically significant improvement in AOFAS but not VAS score at three-month follow-up. Despite not being statistically significant, however, VAS scores were consistently lower in the ESWT group when compared to the control group. In addition, given that AOFAS score consistently improved over three months, and VAS score consistently reduced over three months in both groups, it is possible a more accurate result may have been obtained with a longer follow-up.

Finally, the RCT by Abdelkader et al. [33] found that the addition of ESWT to a stretching and eccentric exercise programme resulted in a statistically significant improvement in VAS and VISA-A scores compared to stretching and eccentric exercises alone. This RCT had the longest follow-up period of 16 months.

Interestingly, the two studies by Rasmussen et al. [30] and Abdelkader et al. [33] were the highest quality RCTs included in this review, based on their ROB and using the GRADE approach [37].

Overall, ESWT appears to be at least as effective as control for the management of midportion Achilles tendinopathy. In addition, it is safe, requires minimal time to administer, and does not require local or regional anaesthesia. It appears from the evidence that a combination of eccentric loading exercises in addition to a course of ESWT may be the most effective intervention. This suggestion is based on the two highest quality studies included in this review [30,33] and has also been suggested in one study of moderate quality [28]. Further high-quality studies with larger sample sizes and involving a combination of treatments is required to determine the most appropriate and effective conservative treatment modality for midportion Achilles tendinopathy. In addition, further studies are required to determine the most effective dose, number of treatments, the time between treatments, and frequency (Hz) of ESWT that should be administered to patients.

Strengths and limitations

The strengths of this paper included the thorough search of the literature, the formal assessment and grading of included studies using validated assessment tools, and the sole inclusion of RCTs, which reduces the ROB in included studies. However, there are also a number of limitations to this review. Firstly, studies that were published in languages other than English were not included which introduces language bias. In addition, there was significant heterogeneity among studies in terms of control interventions, outcomes measures, follow-up, and ESWT protocol which precluded the ability to perform a meta-analysis of results. This may have aided in making recommendations for future research and the incorporation of results into clinical practice. Nevertheless, it should be noted that individual ESWT protocols may be necessary depending on the severity and chronicity of Achilles tendinopathy in addition to patient demands.

## Conclusions

This review suggests that ESWT is a safe and effective modality for treating midportion Achilles tendinopathy. ESWT reduces pain and improves function in those with midportion Achilles tendinopathy. The best available evidence suggests that a combination of ESWT with eccentric exercises and stretching may be even more effective than ESWT alone. Further research is required to confirm this and to determine the optimum ESWT treatment protocol.

Implications for clinical practice

Clinicians should be aware that current evidence from RCTs supports the use of ESWT in the management of midportion Achilles tendinopathy. It remains a safe and effective option, particularly for patients who want to avoid injection therapy or surgery. A combination of ESWT, stretching and eccentric exercises appear to be more effective than ESWT alone.
